# Heart failure induced by cancer therapies: focus on targeted agents, mechanisms, risk prediction, and clinical management

**DOI:** 10.3389/fphar.2026.1766603

**Published:** 2026-02-06

**Authors:** Lifeng Xiao, Xiaoluan Lin, Zhining Yang, Baihan Lin, Renxian Xie

**Affiliations:** 1 Department of Emergency, Cancer Hospital of Shantou University Medical College, Shantou, China; 2 Department of Radiation Oncology, Cancer Hospital of Shantou University Medical College, Shantou, China

**Keywords:** cardio-oncology, cardiotoxicity, heart failure, HER2-targeted therapy, targeted cancer therapy

## Abstract

Targeted therapies have revolutionized oncology but are accompanied by significant cardiovascular complications, with heart failure being a major dose-limiting toxicity. This review primarily focuses on heart failure induced by targeted anticancer agents, while also contextualizing findings with insights from classical chemotherapeutics and radiotherapy where they inform mechanistic understanding or combination regimen management. We detail the multifaceted pathophysiological mechanisms, which vary by drug class, including direct cardiomyocyte injury via HER2/ErbB signaling disruption, mitochondrial dysfunction, oxidative stress, and novel pathways such as ferroptosis and autophagy dysregulation. The review evaluates strategies for risk assessment, highlighting the utility and limitations of clinical tools like Heart Failure Association-International Cardio-Oncology Society (HFA-ICOS) risk score, and acknowledges that while biomarkers and advanced imaging parameters like global longitudinal strain (GLS) are often reported to have high sensitivity for early detection, their performance can vary depending on the specific definitions of cardiotoxicity used and the clinical context. Current management paradigms are discussed, encompassing pharmacological cardioprotection, treatment modification protocols, and the safe continuation of therapy with concomitant cardiac medications. Furthermore, we explore emerging strategies from traditional natural products and gene-based therapies to advanced drug delivery systems aimed at providing targeted cardioprotection. Finally, future perspectives are outlined, focusing on personalized risk prediction through multi-omics and artificial intelligence, and the development of novel therapeutics with improved cardiovascular safety profiles. This mini review underscores the importance of a multidisciplinary cardio-oncology approach to optimize both oncological efficacy and long-term cardiovascular health for cancer patients.

## 1 Introduction

Targeted cancer therapies have revolutionized oncology by selectively inhibiting molecular pathways essential for tumor growth and survival, leading to improved patient outcomes across various malignancies (Chhabra et al., 2025; Gawli et al., 2025; Pamungkas et al., 2025). However, these advances have been accompanied by significant cardiovascular complications, particularly heart failure, which has emerged as a critical limitation in clinical practice (Lang et al., 2025; Nahle et al., 2025). The intersection of oncology and cardiology has given rise to the specialized field of cardio-oncology, focusing on the prevention, detection, and management of cancer therapy-related cardiovascular toxicity (El-Rayes et al., 2025; Wadden et al., 2025).

The spectrum of cardiotoxicity associated with specifically targeted anticancer agents encompasses left ventricular dysfunction, heart failure, hypertension, arrhythmias, and myocardial ischemia (Glen et al., 2025; Lin et al., 2025). These adverse effects not only compromise quality of life but may also necessitate dose reduction or discontinuation of potentially life-saving anticancer treatments (Jin et al., 2025; Yu A. F. et al., 2025). The pathophysiological mechanisms underlying this form of cardiotoxicity are multifaceted, involving direct cardiomyocyte injury, mitochondrial dysfunction, oxidative stress, endothelial damage, and immune-mediated inflammation (Lang et al., 2025; Qing et al., 2025).

Recent evidence suggests that the incidence and manifestations of cardiotoxicity vary considerably among different classes of targeted agents (Budău et al., 2025; Li et al., 2025). HER2-targeted therapies, particularly trastuzumab, are well-documented to cause cardiac dysfunction, though this is often reversible with appropriate management (Taha et al., 2025; Yu A. F. et al., 2025). Tyrosine kinase inhibitors (TKIs), angiogenesis inhibitors, and immune checkpoint inhibitors each present distinct cardiovascular risk profiles (Budău et al., 2025; Gill et al., 2025). The growing arsenal of novel targeted agents, including neurotrophic tyrosine receptor kinase (NTRK) inhibitors such as entrectinib, continues to expand the spectrum of potential cardiovascular complications (Gao et al., 2025).

This review comprehensively examines the current understanding of heart failure associated with cancer therapies, with a primary focus on targeted agents. While anthracyclines and radiotherapy are not molecularly targeted, they are frequently used in combination with targeted therapies or serve as important comparators for cardiotoxicity mechanisms and management principles. Therefore, discussion of anthracyclines and radiotherapy will be included where they provide relevant mechanistic insights or inform broader cardio-oncology management strategies, ensuring a comprehensive perspective on heart failure in the contemporary cancer treatment landscape. By synthesizing evidence from preclinical and clinical studies, we aim to provide a foundation for optimizing cardiovascular care in cancer patients, ultimately enabling the safe and effective delivery of oncological treatments while preserving cardiovascular health.

## 2 Tumor targeted therapy induced heart failure pathophysiological mechanisms

The pathophysiological mechanisms underlying cancer therapy-induced heart failure are complex and multifactorial. While this section details mechanisms pertinent to major classes of targeted agents, insights from classical chemotherapies like anthracyclines are also discussed to provide a comparative mechanistic framework and inform understanding of combination regimens (Lang et al., 2025; Qing et al., 2025). Understanding these drug-specific mechanisms is crucial for developing effective cardioprotective strategies and optimizing the risk-benefit ratio of targeted cancer treatments. This section will detail mechanisms pertinent to major classes of targeted agents.

HER2-targeted therapies, particularly trastuzumab, exert cardiotoxic effects primarily through disruption of ErbB2/ErbB4 signaling in cardiomyocytes (L’Abbate et al., 2025; Pamungkas et al., 2025). This signaling pathway is essential for cardiomyocyte survival, mitochondrial integrity, and protection against oxidative stress. Inhibition of HER2 signaling leads to impaired mitochondrial function, increased susceptibility to apoptosis, and altered cardiac metabolism. Recent evidence suggests that trastuzumab may also induce cardiomyocyte atrophy through fibronectin 1 (FN1)-mediated activation of PI3K/AKT signaling pathways, leading to excessive autophagy and subsequent cellular dysfunction (Zhu et al., 2025).

Tyrosine kinase inhibitors demonstrate diverse cardiotoxicity mechanisms depending on their specific targets. Sorafenib, for instance, promotes oxidative stress and inflammatory responses through modulation of the mercaptopyruvate sulfurtransferase/hydrogen sulfide pathway, exacerbating mitochondrial dysfunction and cellular injury (Salama et al., 2025; Zhong et al., 2025). Sunitinib-induced cardiotoxicity involves activation of MAPK signaling pathways, leading to apoptosis, oxidative stress, and inflammatory responses in cardiomyocytes (Qian and Yi, 2025). Osimertinib, a third-generation EGFR-TKI, has been shown to induce cardiotoxicity through PDK4-mediated mitochondria-endoplasmic reticulum crosstalk, resulting in mitochondrial calcium overload and necroptosis (Deng et al., 2025).

Anthracyclines, though not strictly targeted therapies, are frequently used in combination regimens and warrant discussion due to their profound cardiotoxicity (Fitrianti et al., 2025). It is important to note that while the primary focus of this review is on molecularly targeted agents, anthracyclines are often part of combination regimens with targeted drugs and represent a classic model of severe chemotherapy-induced cardiotoxicity. Their well-characterized mechanisms provide a comparative backdrop and inform combination therapy management. Doxorubicin induces cardiotoxicity through multiple interconnected mechanisms, including topoisomerase IIβ inhibition, oxidative stress, mitochondrial damage, iron metabolism dysregulation, and impaired autophagy (Bhadra et al., 2025; Radeva and Yoncheva, 2025). Recent evidence highlights the role of ferroptosis, an iron-dependent form of regulated cell death, in doxorubicin-induced cardiotoxicity (Bhadra et al., 2025; Han et al., 2025). This process involves glutathione depletion, lipid peroxidation, and disruption of mitochondrial membrane integrity.

Novel mechanisms continue to emerge from recent research. Entrectinib, an NTRK inhibitor, has been found to bind HMGB1 protein at phenylalanine residue 103, enhancing its nuclear localization and subsequently suppressing OTUD5 transcription, which inhibits the MTORC1 pathway and activates autophagy in cardiomyocytes (Gao et al., 2025). This autophagy activation ultimately triggers apoptosis and cardiac dysfunction. Additionally, doxorubicin-treated breast cancer cells secrete small extracellular vesicles (sEVs) enriched with miR-338-3p, which exacerbates doxorubicin-induced ferroptosis in cardiomyocytes by targeting anti-ferroptotic genes including CP, SLC7A11, and GPX4 (Han et al., 2025).

The tumor itself may contribute to cardiotoxicity through secreted factors that render cardiomyocytes more susceptible to chemotherapeutic agents. Inosine and hypoxanthine released by tumor cells activate the A3 receptor on cardiomyocytes, leading to CAMKIIδ phosphorylation and subsequent degradation of the mRNA splicing factor RBFOX1 (Tejay et al., 2025). This degradation reverts cardiomyocytes to a less mature state with open chromatin, increasing their susceptibility to DNA damage and apoptosis when exposed to DNA-intercalating agents.

Environmental factors may also potentiate cardiotoxicity, as demonstrated by 8:2 fluorotelomer alcohol, a persistent environmental pollutant that exacerbates doxorubicin-induced cardiac injury through aryl hydrocarbon receptor activation, promoting mitochondrial dysfunction and AIM2 inflammasome-mediated pyroptosis (Chen et al., 2025).

Understanding these diverse and interconnected, yet often drug-class-specific, pathophysiological mechanisms provides the foundation for developing more precise cardioprotective strategies and personalizing targeted cancer therapy to minimize cardiovascular complications while maintaining antitumor efficacy.

## 3 Heart failure risk assessment and prediction models

Risk assessment and prediction models play a crucial role in identifying patients at increased risk of developing cancer therapy-related cardiac dysfunction (CTRCD), enabling targeted monitoring and preventive strategies (Gomes et al., 2025; Nguyen et al., 2025). The integration of clinical parameters, biomarkers, imaging modalities, and genetic factors has advanced the field toward more personalized risk prediction (Abo Samra et al., 2025; Gomes et al., 2025). It is crucial to distinguish between prediction targets: clinical endpoints such as symptomatic heart failure or cardiovascular death, and surrogate/subclinical endpoints like an asymptomatic decline in left ventricular ejection fraction (LVEF) or global longitudinal strain (GLS). While the latter are valuable for early detection, models predicting them require separate validation against hard clinical outcomes.

The Heart Failure Association-International Cardio-Oncology Society (HFA-ICOS) risk tool represents a significant advancement in CTRCD prediction (Gomes et al., 2025). This tool stratifies patients into low, medium, high, and very high risk categories based on clinical factors including age, cardiovascular risk factors, prior cardiac history, and cancer treatment regimen. However, recent validation studies have revealed limitations in its performance, particularly when mild forms of CTRCD are included as events. In patients with breast cancer receiving anti-HER2 agents, the pooled C statistic for the HFA-ICOS tool was 0.60, indicating modest discrimination ability. Importantly, the tool consistently underestimated risk, with observed event rates exceeding predicted risks across validation studies.

Biomarkers have emerged as valuable components of risk prediction models. Baseline N-terminal pro-B-type natriuretic peptide (NT-proBNP) levels have demonstrated utility in predicting CTRCD in patients receiving BRAF and MEK inhibitors (Glen et al., 2025). Similarly, troponin elevation serves as an early marker of myocardial injury and has been incorporated into several prediction models (Ishii et al., 2025; Ju et al., 2025; Omland et al., 2025). Emerging biomarkers like soluble suppression of tumorigenicity-2 (sST2), which reflects cardiac fibrosis and remodeling, show promise. A systematic review and meta-analysis indicated dynamic changes in sST2 during cardiotoxic cancer treatment, suggesting its potential role in risk stratification and early detection (Fazzini et al., 2025a). A prospective study of breast cancer patients receiving trastuzumab identified prolonged QTc interval (>450 ms) and lower expression levels of estrogen and progesterone receptors as significant predictors of cardiotoxicity (Abo Samra et al., 2025). These findings highlight the potential integration of electrocardiographic parameters and tumor characteristics into risk assessment frameworks.

Echocardiographic parameters, particularly GLS, have shown promise in improving risk prediction beyond conventional ejection fraction measurements (Nguyen et al., 2025). In patients receiving anthracyclines or trastuzumab, relative decline in LV-GLS >15% demonstrated excellent predictive performance for CTRCD, with area under the curve values of 0.93 and 0.97 for anthracycline and trastuzumab-related cardiac dysfunction, respectively. However, it is important to note that a strain-guided management strategy has not consistently translated into improved clinical outcomes in all settings. The SUCCOUR trial, a randomized controlled trial comparing strain-guided to ejection fraction-guided management of potentially cardiotoxic therapy, did not show a significant difference in the primary endpoint of change in LVEF at 3-year follow-up, highlighting the complexity of translating imaging surrogates into patient-centric benefit (Negishi et al., 2023). The addition of baseline HFA-ICOS risk scores did not further improve the predictive performance of GLS monitoring.

Advanced imaging modalities are increasingly incorporated into risk assessment protocols. Comprehensive cardiovascular assessment including stress perfusion cardiovascular magnetic resonance imaging and blood biomarkers has been implemented in prospective studies of patients receiving BRAF and MEK inhibitors (Glen et al., 2025). Radiomics analysis of baseline echocardiography images using machine learning algorithms has demonstrated impressive accuracy in predicting post-chemotherapy cardiotoxicity, with K-nearest neighbors and linear support vector machine models achieving accuracies of 0.92 and 0.90, respectively, for short-axis views (Ahmadi et al., 2025).

Genetic factors contribute significantly to individual susceptibility to CTRCD. Pharmacogenomic studies have identified polymorphisms in drug metabolism-related genes that influence anthracycline-induced cardiotoxicity risk (Vaitiekus et al., 2025). Furthermore, comprehensive reviews highlight the evolution from candidate gene studies to polygenic risk scores in understanding the genetic architecture of heart failure, including therapy-induced forms, underscoring the potential for genetic stratification in cardio-oncology (Figueiral et al., 2024). From a clinical perspective, identifying genetic predisposition in cancer patients can inform personalized surveillance and prevention strategies (Farmakis et al., 2023). Recent studies continue to characterize the genetic background in patients who develop cancer therapy-induced cardiomyopathy, reinforcing its polygenic nature (Fazzini et al., 2025b). Landmark genome-wide association studies have identified specific genetic variants associated with increased risk of cancer therapy-induced cardiomyopathy, such as those in CELF4 and HTR2C, providing insights into pathophysiological pathways (Garcia-Pavia et al., 2019).

Beyond genetics, metabolomic profiling offers a complementary approach to understand cardiotoxicity. Studies analyzing metabolic shifts have identified potential early diagnostic signatures and shed light on disrupted energetic pathways in anthracycline-induced cardiotoxicity, opening avenues for novel biomarker discovery (Fazzini et al., 2022; Singh et al., 2025). The complex polygenic nature of CTRCD susceptibility underscores the need for comprehensive genetic profiling in risk prediction (Solomon et al., 2025).

Despite these advances, significant challenges remain in CTRCD prediction. Most existing models demonstrate high risk of bias, limited external validation, and poor reporting of key performance metrics (Gomes et al., 2025). Nearly all developed models were at high risk of bias, and only 24% underwent external validation. A critical gap is the scarcity of prediction models specifically developed and validated for the broad spectrum of targeted therapy-induced cardiotoxicity. Most existing tools focus primarily on anthracycline- or HER2-targeted therapy-related cardiotoxicity, highlighting an unmet need for models encompassing newer targeted agents such as TKIs, angiogenesis inhibitors, and immune checkpoint inhibitors.

Future directions in CTRCD risk assessment include the integration of multi-omics approaches, artificial intelligence-assisted analysis of multimodal data, and development of dynamic risk prediction models that incorporate changes in parameters during treatment (Solomon et al., 2025). The evolving landscape of cancer therapeutics necessitates continuous refinement of risk prediction tools to address the cardiovascular effects of novel targeted agents and combination regimens.

**FIGURE 1:**
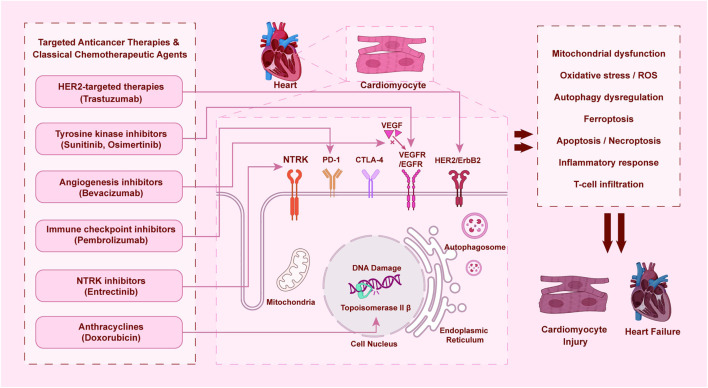
Mechanisms of cardiotoxicity induced by targeted anticancer therapies and classical chemotherapeutic agents.

The diverse yet interconnected mechanisms of cardiotoxicity induced by major classes of targeted anticancer agents and relevant chemotherapeutics are summarized in Figure 1. This schematic provides a comparative overview of key drug classes, their primary molecular targets, and the downstream cellular events leading to cardiomyocyte injury and heart failure, highlighting both shared and distinct pathological pathways.

## 4 Heart failure monitoring and diagnostic strategies

Comprehensive monitoring and accurate diagnosis of heart failure in patients receiving targeted cancer therapies are essential for early intervention and optimal management. Current strategies encompass multimodal approaches including biochemical biomarkers, advanced imaging techniques, and functional assessments (Omland et al., 2025).

Biomarkers play a central role in the surveillance of cardiotoxicity. Cardiac troponins (cTnI and cTnT) serve as sensitive indicators of myocardial injury and have demonstrated prognostic value in various cancer therapy settings (Omland et al., 2025). Natriuretic peptides (BNP and NT-proBNP) reflect ventricular wall stress and have utility in detecting early cardiac dysfunction (Yu S. et al., 2025). In patients receiving BRAF and MEK inhibitors, elevated baseline NT-proBNP levels were associated with increased risk of cancer therapy-related cardiac dysfunction (Glen et al., 2025). Emerging biomarkers including sST2 and galectin-3 show promise in enhancing risk stratification and early detection of cardiotoxicity (Gomaa et al., 2025; Quagliariello et al., 2025). sST2, in particular, has been highlighted in reviews as a stable biomarker less influenced by age and renal function than natriuretic peptides, with potential utility in both risk prediction and monitoring of therapeutic response in CTRCD, though its integration into routine practice requires further standardization (Murtagh et al., 2023; Negishi et al., 2023).

Echocardiography remains the cornerstone of cardiac monitoring during cancer therapy. LVEF measurement continues to be the primary parameter for defining cardiotoxicity, with current guidelines defining cancer therapy-related cardiac dysfunction as a reduction in LVEF of >10 percentage points to a value below 50% (Nguyen et al., 2025). However, LVEF has limitations as a monitoring tool due to its relatively low sensitivity for detecting early myocardial damage.

Myocardial strain imaging, particularly GLS, has emerged as a more sensitive technique for detecting subclinical ventricular dysfunction. Studies have demonstrated that changes in GLS often precede LVEF reduction and may identify patients at risk of developing overt cardiotoxicity. In patients receiving anthracyclines or trastuzumab, a relative decline in LV-GLS >15% demonstrated excellent predictive performance for subsequent CTRCD (Nguyen et al., 2025). The Tei index (myocardial performance index) has also shown utility in early detection of subclinical cardiac dysfunction, with an area under the curve of 0.867 for predicting cardiotoxicity (Ju et al., 2025).

Advanced cardiac imaging modalities offer additional insights into myocardial structure and function. Cardiovascular magnetic resonance (CMR) provides precise quantification of ventricular volumes and function, tissue characterization through late gadolinium enhancement and parametric mapping, and assessment of myocardial inflammation (Glen et al., 2025; Lu K. et al., 2025). Stress perfusion CMR can identify microvascular dysfunction in patients receiving targeted therapies. In prospective studies of patients receiving BRAF and MEK inhibitors, comprehensive cardiovascular assessment including CMR has been integrated into monitoring protocols.

The frequency and duration of cardiac monitoring should be individualized based on patient risk profile and cancer treatment regimen. Current guidelines typically recommend echocardiographic monitoring every 3 months during HER2-targeted therapy. However, emerging evidence suggests that reduced surveillance frequency may be considered in selected low-risk populations (Yu A. F. et al., 2025). A recent non-randomized clinical trial evaluating reduced CTRCD surveillance performed every 6 months specifically in patients receiving non-anthracycline HER2-targeted therapy reported a low incidence of cardiac events, with no symptomatic events at 1 year and only one case of asymptomatic CTRCD in this specific study cohort. This finding suggests the feasibility of a less frequent monitoring strategy in this particular context, although the non-randomized design and selective population warrant caution in generalizing this approach.

Novel monitoring approaches are under investigation to enhance early detection of cardiotoxicity. Radiomics analysis of echocardiography images using machine learning algorithms has demonstrated impressive accuracy in predicting post-chemotherapy cardiotoxicity (Ahmadi et al., 2025). The development of theranostic probes, such as hydrogen peroxide-responsive fluorescent probes derived from tanshinone IIA, enables simultaneous monitoring and treatment of doxorubicin-induced cardiotoxicity (Cao et al., 2025). These innovative approaches may facilitate personalized monitoring strategies based on individual risk profiles.

Electrocardiographic monitoring provides important information on electrical abnormalities associated with targeted therapies (Lin et al., 2025). QTc prolongation is a well-described adverse effect of several tyrosine kinase inhibitors and requires regular monitoring. Baseline QTc prolongation and history of cardiovascular disease were identified as the most significant contributors to QTc prolongation risk.

The integration of multimodal monitoring data through artificial intelligence and machine learning approaches holds promise for enhancing early detection and risk stratification. However, challenges remain in standardizing monitoring protocols, defining appropriate response thresholds, and determining the clinical implications of subfunctional changes detected through advanced imaging techniques.

The primary objective of structured monitoring extends beyond the identification of isolated imaging abnormalities. Its critical role lies in linking surrogate markers, such as LVEF and GLS, to the tangible clinical outcomes of heart failure that impact patient survival and oncology care—namely, the development of symptomatic heart failure, unplanned cardiovascular hospitalizations, premature discontinuation of effective anticancer therapy, and cardiac death. Therefore, the interpretation of any diagnostic finding must be contextualized within this clinical continuum. A decline in GLS, while subclinical, signifies elevated risk for subsequent overt dysfunction and should prompt preemptive cardioprotective strategies. Conversely, a confirmed drop in LVEF below 50%, especially if accompanied by biomarker elevation, moves the patient along this continuum towards a state requiring direct intervention on the cancer treatment regimen. By framing monitoring results as waypoints along the path to possible clinical events, surveillance transitions from a passive list of tests to an active tool for risk stratification and timely intervention, thereby directly informing the subsequent management decisions.

## 5 Heart failure prevention and management strategies

Effective prevention and management of therapy-induced heart failure require a nuanced approach that prioritizes interventions based on the strength of supporting evidence. We propose a stratified framework to guide clinical decision-making: (1) guideline-supported standard practice, (2) emerging strategies with promising but incomplete evidence, and (3) experimental or preclinical strategies. This framework must be integrated with clear protocols for practical management decisions, including when to continue or interrupt cancer therapy, the timing of cardiology referral, and schedules for cardiac reassessment.

Guideline-supported standard practice forms the cornerstone of management and is centered on a tiered clinical pathway. For high-risk patients before or during therapy, primary prevention with angiotensin-converting enzyme inhibitors, angiotensin II receptor blockers, and/or beta-blockers is recommended (Fitrianti et al., 2025). This recommendation is supported by systematic reviews and meta-analyses confirming their role in preventing cardiac dysfunction during anthracycline and HER2-targeted therapy (Sotiropoulou et al., 2024). Upon detection of subclinical myocardial injury, immediate initiation or optimization of these cardioprotective drugs is required, alongside a formal cardio-oncology consultation to decide on modifying the cancer treatment plan. For overt cancer therapy-related cardiac dysfunction, management is anchored in initiating standard guideline-directed medical therapy for heart failure, and a critical decision must be made regarding the necessity of holding or discontinuing the causative anticancer agent.

Emerging strategies encompass interventions with encouraging yet not fully conclusive data, often supported by single randomized trials, robust preclinical data, or extensive observational studies. This category includes the use of sacubitril/valsartan for cardioprotection, which preserved myocardial strain in a clinical trial, and the potential application of statins, whose pleiotropic effects are supported by mechanistic studies but require further confirmation in dedicated cardio-oncology trials (Omland et al., 2025; TamehriZadeh et al., 2025). Non-pharmacological strategies, such as structured exercise programs and specific micronutrient supplementation (Konstantinidis et al., 2025; Nsairat et al., 2025), also reside here, with evidence primarily from preclinical models or small clinical studies (Zhao et al., 2025). Recent comprehensive reviews summarize the translational evidence and current potential of these evolving pharmacological and non-pharmacological approaches (Avagimyan et al., 2025; Migliari et al., 2025). Practical decisions involving these strategies should be individualized, often within a multidisciplinary team or clinical trial setting.

Experimental and preclinical strategies represent the frontier of cardio-oncology research, offering future therapeutic possibilities but currently lacking direct clinical validation. This broad category includes novel compounds derived from natural products and traditional medicines (such as specific flavonoids, berberine, herbal formulations), which have demonstrated multi-target protective effects in cellular and animal models of cardiotoxicity (Chou et al., 2025; Sheng et al., 2025). It also encompasses advanced biotechnology approaches such as gene-editing techniques, sophisticated nanoparticle-based drug delivery systems designed for tumor-specific targeting, and novel immunomodulatory agents (Lee et al., 2025). While not yet ready for routine clinical application, these strategies provide critical insights into disease mechanisms and hold promise for future targeted interventions.

Integral to applying this stratified evidence framework are clear, actionable protocols for key management decisions. The critical choice of continuing versus interrupting life-saving cancer therapy must be guided by the severity of cardiac dysfunction, its reversibility, and the availability of alternative anticancer regimens. Early formal cardio-oncology referral is recommended at the stage of risk stratification and is mandatory upon detection of any cardiac abnormality. The frequency of cardiac reassessment, typically via echocardiography and biomarkers, must be individualized; it may follow standard intervals for low-risk patients but requires intensification following any change in cardiac status or therapy. Ultimately, managing heart failure in cancer patients is an exercise in balancing dual risks, requiring seamless collaboration between oncology and cardiology to optimize overall patient outcomes. This outcomes-oriented framework ensures that surveillance and intervention are directly linked to preserving both oncologic efficacy and cardiovascular health. The key pathophysiological mechanisms and the stratified prevention/management strategies discussed are summarized in Tables 1, 2, respectively.

**TABLE 1:** Key mechanisms of heart failure induced by major classes of targeted anticancer agents and relevant contextual chemotherapies.

Therapeutic class	Representative agents	Primary oncologic target	Key proposed cardiotoxic mechanisms
HER2-targeted therapies	Trastuzumab, Pertuzumab	HER2/ErbB2 receptor	Disruption of ErbB2/ErbB4 survival signaling mitochondrial dysfunction; increased apoptosis; induction of cardiomyocyte atrophy via excessive autophagy
Tyrosine kinase Inhibitors(TKls)			
Multikinase inhibitors	Sunitinib, Sorafenib	VEGFR, PDGFR,RAF,KIT	Oxidative stress; inflammatory response; mitochondrial dysfunction; MAPK pathway-mediated apoptosis
EGFR inhibitors	Osimertinib	EGFR (T790M)	Mitochondria-ER crosstalk dysfunction leading to calcium overload and necroptosis
Angiogenesis inhibitors	Bevacizumab	VEGF	Endothelialdamage; hypertension; myocardial ischemia
Immune checkpoint inhibitors	Pembrolizumab, Ipilimumab	PD-1,CTLA-4	Immune-mediated myocarditis; T-cell driven myocardial inflammation and necrosis
NTRK inhibitors	Entrectinib	NTRK1/2/3	HMGB1 binding and subsequent induction of excessive autophagic flux in cardiomyocytes
Contextual Agent:Anthracyclines	Doxorubicin	Topoisomerase IIB (non-specific)	Topoisomerase IIβ inhibition; massive ROS generation; mitochondrial damage; iron dysregulation andferroptosis

Abbreviations: CTRCD, Cancer therapy-related cardiac dysfunction; HER2, Human epidermal growth factor receptor 2; EGFR, epidermal growth factor receptor; VEGFR, vascular endothelial growth factor receptor; PDGFR, Platelet-derived growth factor receptor; RAF, rapidly accelerated fibrosarcoma; KIT, Mast/stem cell growth factor receptor; NTRK, neurotrophic tyrosine receptor kinase; VEGF, vascular endothelial growth factor; PD-1, Programmed cell death protein 1; CTLA-4, Cytotoxic T-lymphocyte-associated protein 4; HMGB1, High mobility group box 1; MTORC1, Mechanistic target of rapamycin complex 1; MAPK, Mitogen-activated protein kinase; ER, endoplasmic reticulum; ROS, reactive oxygen species; sEVs, small extracellular vesicles.

**TABLE 2:** Stratified interventions for the prevention and management of cancer therapy-related cardiac dysfunction (CTRCD).

Category and Strategy	Specific Interventions/Approaches	Evidence Level and Key Considerations
Guideline-Supported Standard		
Primary pharmacological prophylaxis	ACE inhibitors (lisinopril), ARBs (valsartan), beta-blockers (carvedilol)	Standard for high-risk patients (such as per HFA-ICOS score) receiving anthracyclines± HER2-targeted therapy Supported by meta-analyses
Management of overt CTRCD/heart failure	Guideline-directed medical therapy for heart failure (GDMT)	Mandatory upon diagnosis of symptomaic heart failure or significant LVEF drop.May necessitate cancer therapy interruption
Emerging strategies (incomplete evidence)		
Advanced neurohormonal Blockade	Sacubitril/valsartan	RCT data shows benefit on biomarkers and GLS, but not on primary LVEF outcome. Role in prophylaxis under investigation
Adjunctive pharmacotherapy	Statins (atorvastatin)	Strong preclinical and observational data; conclusive RCT evidence in cardio-oncology pending
Non-pharmacological interventions	Structured aerobic exercise programs	Consistent benefit in preclinical models; promising clinical data supports integration into comprehensive care
Experimental/Preclinical strategies		
Natural product Derivatives	Astragaloside IV, tanshinone IIA, Qishen Huanwu capsule	Multi-target cardioprotection shown *in vitro* and in animal models. Clinical translation requires validation
Targeted molecular agents	HMGB1 inhibitors, CISD2 activators, ferroptosis inhibitors	Mechanism-specific, designed based on drug toxicity profiles. In early-stage translational research
Advanced drug delivery	Tumor-targeted liposomes, stimuli-responsive nanoparticles	Aim to reduce cardiac drug exposure. Several platforms in preclinical or early clinical development

Abbreviations: CTRCD, Cancer therapy-related cardiac dysfunction; HFA-ICOS, Heart Failure Association-International Cardio-Oncology Society; GLS, global longitudinal strain; LVEF, left ventricular ejection fraction; ACEI, Angiotensin-converting enzyme inhibitor; ARB, Angiotensin II, receptor blocker; GDMT, Guideline-directed medical therapy; RCT, randomized controlled trial; PFS, Progression-free survival; OS, overall survival; AEs, Adverse events; QoL, quality of life.

## 6 Emerging therapeutic strategies and future perspectives

The evolving landscape of cardio-oncology is driving innovation toward more precise and effective cardioprotection. To maintain focus on transformative potential, this section highlights three high-impact future directions poised to reshape management: precision risk stratification, targeted cardioprotection, and smart drug delivery systems.

### 6.1 Precision risk stratification via multi-omics and AI

Future risk assessment will move beyond clinical scores by integrating multi-omics data (genomics, proteomics, metabolomics) with artificial intelligence. Human induced pluripotent stem cell (hiPSC)-derived cardiomyocyte models facilitate the identification of genetic variants linked to drug-induced toxicity, enabling personalized risk profiling and *in vitro* drug screening (Solomon et al., 2025) Machine learning algorithms applied to multimodal data—including serial biomarkers, advanced imaging radiomics, and genetic profiles—will enable dynamic prediction of cardiotoxicity, shifting from static baseline assessment to real-time, adaptive risk monitoring.

### 6.2 Targeted cardioprotection based on mechanism

Emerging strategies aim to intercept specific molecular pathways of cardiotoxicity without compromising oncologic efficacy. This includes inhibition of cardiotoxic off-targets (such as HMGB1 inhibition for entrectinib), activation of endogenous protective pathways (CISD2 activation), and modulation of regulated cell death programs like ferroptosis and pyroptosis (Chou et al., 2025; Gao et al., 2025; Han et al., 2025). Natural product-derived compounds (e.g., astragaloside IV, tanshinone IIA) and formulations from traditional medicine, with their multi-target profiles, offer rich scaffolds for developing such targeted adjuvants (Lv et al., 2025; Yu X. et al., 2025).

### 6.3 Advanced and smart drug delivery systems

Redesigning anticancer drugs themselves to minimize cardiac exposure is a key frontier. Innovations include liposomal formulations of anthracyclines, albumin-bound nanoparticles, and tumor microenvironment-responsive prodrugs (Hao et al., 2025; Lu J. et al., 2025). Next-generation “smart” systems utilize ligands for active tumor targeting and stimuli-responsive linkers for controlled release, dramatically enhancing tumor-specific drug accumulation while sparing the heart and other healthy tissues (Lee et al., 2025; Han et al., 2026). These approaches decouple antitumor efficacy from cardiotoxicity at the pharmacokinetic and pharmacodynamic levels.

The synergy of these directions points toward a future of personalized cardio-oncology. Precision stratification will identify who needs protection, targeted adjuvants will provide mechanism-specific shielding, and smart drug delivery will minimize the need for broad cardioprotection by reducing cardiac insult at the source. Importantly, technological advances in radiotherapy (Xie et al., 2024) (such as proton therapy, deep-inspiration breath-hold) complement these pharmacological strategies by minimizing incidental cardiac dose, exemplifying how multidisciplinary engineering enhances overall cardiovascular safety. The ultimate goal is an integrated “prevention-monitoring-treatment” system, ensuring that progress in cancer survivorship is not offset by cardiovascular morbidity.

**FIGURE 2:**
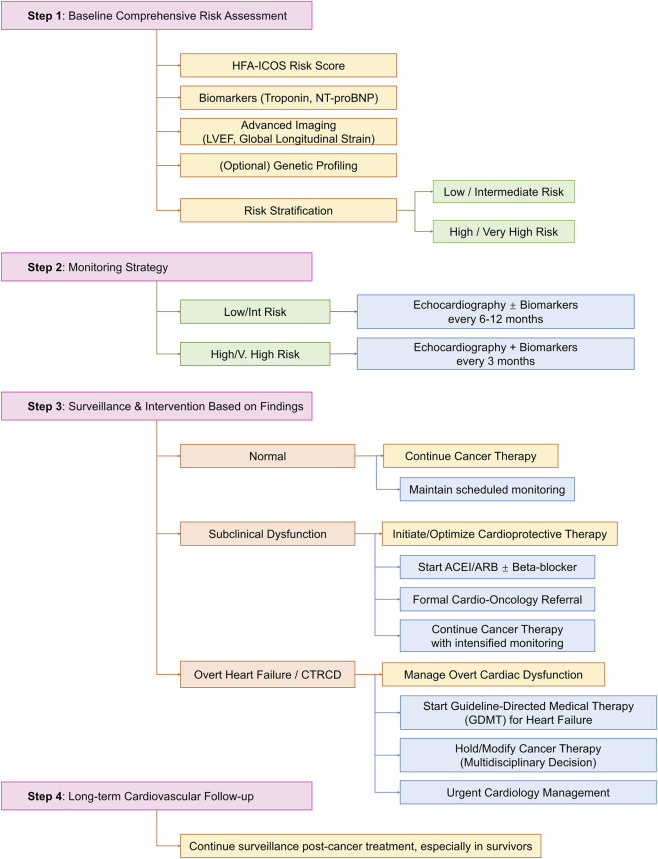
Clinical management algorithm for cancer therapy-related cardiac dysfunction (CTRCD).

A structured, evidence-based approach to the prevention and management of CTRCD is essential for balancing oncologic efficacy and cardiovascular safety. Figure 2 outlines a comprehensive clinical algorithm integrating risk assessment, monitoring, and stratified interventions, from primary prevention in high-risk patients to the management of overt heart failure.

## 7 Conclusion and outlook

The management of heart failure associated with cancer therapy, particularly targeted agents, has evolved from a dose-limiting complication to a manageable condition. This progress has been driven by advancements in understanding drug-specific mechanisms, the development of structured risk assessment and monitoring protocols, and the implementation of evidence-based cardioprotective strategies. The establishment of multidisciplinary cardio-oncology programs is central to optimizing cardiovascular care, enabling safer delivery of effective cancer treatments.

Key to this evolution is the shift towards personalized management. Risk prediction now integrates clinical, biomarker, imaging, and genetic data to identify vulnerable patients. Monitoring has been refined with sensitive tools like global longitudinal strain for early detection, allowing for timely intervention. Management strategies are increasingly tailored, ranging from primary pharmacoprevention in high-risk individuals to guideline-directed heart failure therapy in symptomatic patients, with careful consideration of anticancer treatment modification.

Despite significant progress, challenges remain, including the need for dynamic risk models for novel therapies, validation of advanced biomarkers, and the translation of promising preclinical cardioprotective strategies into clinical practice. Future efforts must focus on integrating multi-omics and artificial intelligence for precision management, developing targeted cardioprotectants, and advancing drug delivery systems to enhance tumor specificity. Continued collaboration across oncology, cardiology, and translational research is essential to ensure that improvements in cancer survival are matched by the preservation of long-term cardiovascular health.
